# Metabolic Dysregulation in Alzheimer's Disease: A High‐Resolution Brain Metabolomics Approach

**DOI:** 10.1002/alz70856_104496

**Published:** 2025-12-25

**Authors:** Donghai Liang, Youran Tan, Emma Casey, Zhenjiang Li, Marla Gearing, Allan I. Levey, James J. Lah, Aliza P. Wingo, Thomas S. Wingo, Dean P Jones, Douglas I Walker, Anke Huels

**Affiliations:** ^1^ Emory University, Atlanta, GA, USA; ^2^ Goizueta Alzheimer's Disease Research Center, Emory University, Atlanta, GA, USA; ^3^ Emory University School of Medicine, Atlanta, GA, USA; ^4^ Goizueta Alzheimer's Disease Research Center, Emory University, Atlanta, GA, USA; ^5^ University of California, Davis, Sacramento, CA, USA; ^6^ UC Davis School of Medicine, Sacramento, CA, USA; ^7^ Department of Medicine, Emory University, Atlanta, GA, USA

## Abstract

**Background:**

Alzheimer's disease (AD) is a complex neurodegenerative disorder characterized by progressive cognitive decline and neuropathological hallmarks. Despite extensive research, the molecular mechanisms linking metabolic dysregulation to AD neuropathology remain poorly understood. Metabolomics, a powerful tool for profiling small molecules, provides an opportunity to identify metabolic signatures and pathways implicated in disease progression. To address this gap, we conducted a comprehensive high‐resolution brain metabolomics study, profiling metabolic perturbations associated with AD neuropathology.

**Method:**

Using untargeted high‐resolution metabolomics, we analyzed 162 frontal cortex samples from the Emory Goizueta Alzheimer's Disease Research Center brain bank with comprehensive neuropathological evaluations, including Braak stage, CERAD, and ABC scores. We conducted a metabolome‐wide association study of AD neuropathology adjusting for confounders and multiple testing (FDR 5%). Pathway enrichment analysis was performed to uncover biological processes implicated in AD, and chemical annotation confirmed key metabolites with Level 1 evidence. We also examined potential effects of APOE ε4 genotype in modifying the associations between significant metabolic features and AD neuropathology markers.

**Results:**

Our analysis identified 155 metabolic features, and 36 metabolic pathways significantly associated with the AD neuropathology markers, spanning ten metabolic classes such as energy metabolism, nucleotide metabolism, and amino acid metabolism. ABC score was linked to the largest number of pathways, while Braak stage and CERAD exhibited distinct metabolic associations, including nucleotide metabolism with tau pathology and fatty acid‐related pathways with neuritic plaque deposition. Further, 18 unique metabolites were confirmed with level 1 evidence, implicating their involvement in amino acid metabolism, lipid metabolism, carbohydrate metabolism, nucleotide metabolism, and metabolism of cofactors and vitamins in AD neuropathology. Genetic variability influenced these associations, with non‐carriers of the APOE ε4 allele showing stronger perturbations in metabolites including glucose, mannose, myo‐inositol, and adenosine 5'‐diphosphoribose.

**Conclusion:**

This study demonstrates the potential of high‐resolution metabolomic profiling in brain tissues to elucidate molecular mechanisms underlying AD pathology. Our findings provide critical insights into metabolic dysregulation in AD and its interplay with genetic factors. Future longitudinal studies are needed to validate these findings and explore the functional significance of the identified metabolites in AD progression.

